# The biological function and research progress of the adipokine chemerin in tumorigenesis and development

**DOI:** 10.7150/jca.117040

**Published:** 2025-08-11

**Authors:** Yongshuai Jiang, Yingying Wei, Ziyang Li, Junsheng Dong, Weijuan Gong

**Affiliations:** 1 Guangling College, Yangzhou University, Yangzhou 225009, China.; 2 Department of Immunology, School of Medicine, Yangzhou University, Yangzhou 225009, China.; 3 College of Veterinary Medicine, Yangzhou University, Yangzhou 225009, Jiangsu, China.

**Keywords:** Chemerin, Chemerin receptor, Tumor microenvironment, Molecular therapy

## Abstract

Chemerin is a protein, encoded by the *RARRES2* gene, which has important roles in immune regulation, inflammation and metabolic regulation. Chemerin can affect the proliferation, migration and invasion ability of tumor cells and is important in the occurrence, development, metastasis, differentiation and development of tumors. CMKLR1, GPR1, and CCRL2, the primary cellular receptors for chemerin, can be found in both normal and tumor tissues. Chemerin binds to its receptors to influence tumor growth and metastasis by regulating the inflammatory response and tumor microenvironment. In this paper, the mechanism of chemerin and its receptors in the tumor microenvironment was summarized, providing theoretical basis for further study of the mechanism of chemerin in tumors and for molecular targeted therapy based on chemerin.

## 1. Introduction

Chemerin, also known as retinoic acid receptor reactive protein 2 (*RARRES2*) or tazarotene-inducing gene 2 (*TIG2*), is a new adipocytokine released after adipocyte maturation that plays a role in inflammation, lipogenesis and glucose homeostasis 1, 2. Chemerin may influence tumor growth by modulating immune cell infiltration and inflammatory responses in the tumor microenvironment. It may also directly affect tumor growth by affecting the proliferation, apoptosis and metastasis of tumor cells. Many studies have shown that chemerin has dual effects on tumors: an antitumor effect on some tumors and a stimulative effect on others 3, 4. The exact mechanism of action of chemerin in tumorigenesis and development remains to be clarified. The interactions between chemerin and immune cells in the context of tumor initiation, progression, and metastasis highlight the complex interplay between the immune system and the tumor microenvironment. Targeting these interactions may offer potential therapeutic strategies for cancer treatment by modulating the immune response within the tumor microenvironment.

Chemerin is a newly discovered adipokine, mainly secreted by adipose tissue in the form of autocrine or paracrine means playing an important role in cell physiology and pathology. In a variety of tumors, the abnormal expression of chemerin is associated with the occurrence, development, invasion and metastasis of tumors. Chemerin binds to the receptors CMKLR1, GPR1 or CCRL2 to recruit immune cells, which then regulate the immune inflammatory response of the body and assume antitumor and protumor roles in tumor progression through interactions with different pathways. In-depth study of the mechanism of chemerin and its regulated target genes will be helpful for exploring potential biomarkers of tumors and identifying new targets for the clinical treatment of tumors.

## 2. Structure and Biological Function

**Figure 1:**
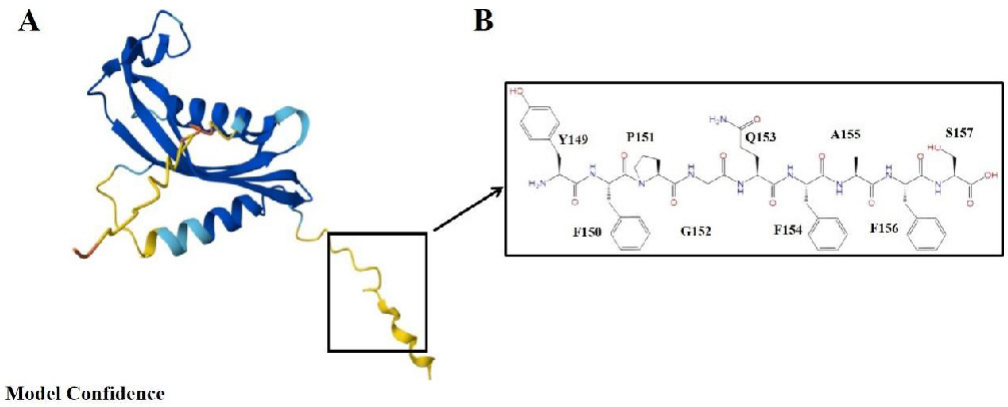
** The 3D model of chemerin and structure of C-terminal nonapeptide. (A)** The AlphaFold predicted structure of chemerin (AF-A0A090N7U9-F1), pLDDT represents per-residue model confidence score between 0 and 100; **(B)** Chemerin nonapeptide equivalent to amino acid 149-157.

Chemerin, a small molecular protein composed of positive alkaline amino acids, was originally discovered to be a cytokine produced by fat cells and is widely expressed in human tissues, especially in tissues such as fat, liver, lungs, pancreas, intestine, and skin, indicating its involvement in a variety of physiological and pathological processes. Human chemerin is a protein consisting of 163 amino acids, and its N-terminal signal sequence of 20 amino acids is removed before it is secreted into the bloodstream by a C-terminal process. The precursor form of chemerin, an 18kDa preprotein with weak activity, was initially regarded as a chemical chemoattractant. Following enzymatic proteolytic hydrolysis it attracts cells involved in innate and adaptive immune responses and transforms them into a novel state that plays a crucial role in adipogenesis and chemotaxis within the innate immune system 5. The biologically active chemerin (chemerin 21-157) results from proteolytic cleavage of prochemerin and uses its C-terminal peptide containing the sequence YFPGQFAFS for receptor activation, which represents a structural basis for chemerin recognition by CMKLR1 (human chemerin receptor 1) for the established chemotactic and adipokine activities 6,7 (Figure 1).

**Figure 2:**
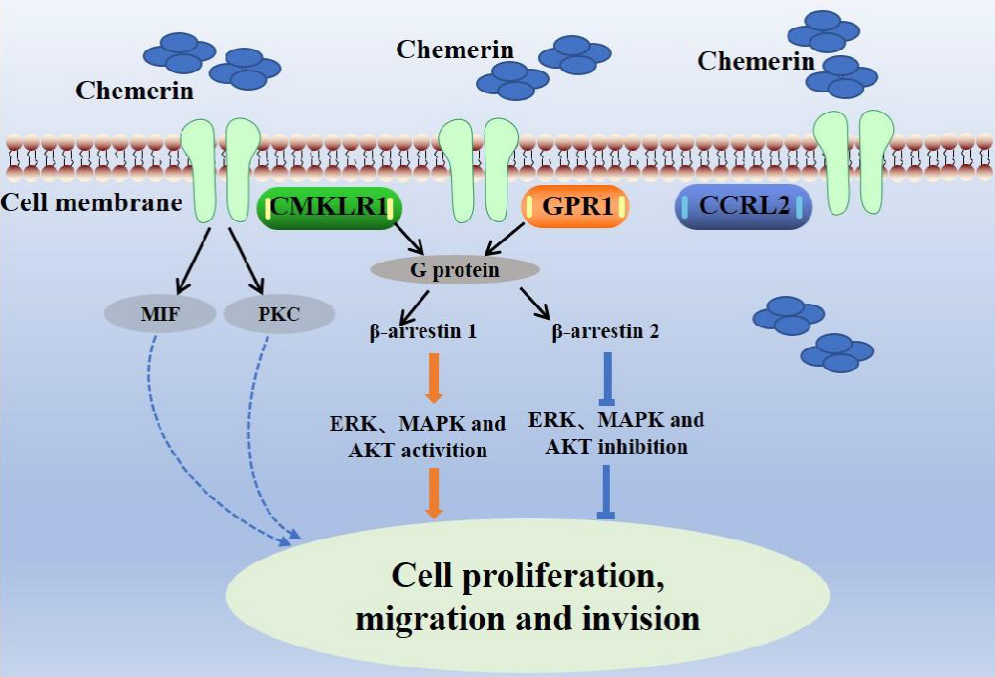
Interactions between chemerin and chemerin receptors affect cell function.

Chemerin has three main receptors: chemokine-like receptor-1 (CMKLR1, ChemR23), G-protein-coupled receptor-1 (GPR1), and chemokine (C-C motif) receptor-like 2 (CCRL2) 8,9. ChemR23 is a G-protein-coupled receptor expressed on monocytes, macrophages, dendritic cells, and natural killer cells 10. Chemerin binds to ChemR23 to cause dendritic cells and macrophages to enter inflammatory tissue. Chemerin/ChemR23 activates the ERK, MAPK and AKT signaling pathways to promote the expression of IL-6 and TNF-α 11. In addition, Chemerin/ChemR23 binding triggers intracellular signaling processes related to calcium ion concentration, cyclic adenosine phosphate (cAMP), and protein kinase activity 12. GPR1, also a G-protein-coupled receptor, was first identified as an orphan receptor in the human hippocampus in 1994, and chemerin was reported as a natural ligand of GPR1 in 2008 13. GPR1 is the closest homolog of CMKLR1, sharing more than 40% sequence homology with CMKLR. GPR1 is expressed in the central nervous system, skin, adipose tissue and granulosa cells. The binding of chemerin and chemerin-9 peptides induces the recruitment of β-arrestin 1 and β-arrestin 2 to CMKLR1 and GPR1 to varying degrees 14. CCRL2 is a seven-pass transmembrane receptor that does not activate conventional G-protein-dependent signaling or induce directed cell migration. CCRL2 has no known downstream signal, presenting to CMKLR1 and GPR1 to affect the local activity of chemerin 15. The combination of chemerin and CCLR2 induces leukocyte chemotaxis and the expression of CCRL2 in tumor cells can increase the local activity of chemotherapy drugs, inhibit neovascularization, and subsequently inhibit tumor growth 9. The interactions between chemerin and its receptors affect cell function (Figure 2).

Chemerin performs its biological function by binding to its receptors. These receptors are widely expressed in immune cells and other cell types. Blood chemerin levels are elevated in patients with inflammatory bowel disease, cardiovascular disease, multiple sclerosis, obesity, and early type 2 diabetes 16-20. Chemerin is produced primarily by adipocytes, which interact with cancer cells, immune cells, fibroblasts, and endothelial cells and coordinate multiple signaling pathways by secreting bioactive molecules, including adipokines 21. Recent studies have shown that patients with colorectal cancer (CRC) have increased levels of chemerin in their blood and that these levels increase as the tumor, node, and metastasis (TNM) stage of the disease progresses. The biological functions of chemerin include the following: (1) Inflammation regulation: Chemerin plays an important role in the inflammatory response, can recruit and regulate the migration and activation of immune cells, and can participate in the regulation of inflammatory processes 22. (2) Metabolic regulation: Chemerin is associated with metabolic processes such as glucose metabolism, lipid metabolism, and insulin sensitivity. It may play a role in the pathogenesis of diseases such as obesity, diabetes and metabolic syndrome 23. (3) Immune regulation: Chemerin can affect the function of immune cells, such as regulating the activity of T cells, dendritic cells and macrophages 24. Chemerin is a potent chemotactic protein that promotes the recruitment of immune cells such as monocytes/macrophages, neutrophils, natural killer cells, and dendritic cells 25.

In conclusion, chemerin acts as a cytokine that plays important roles in immune regulation, inflammation, and metabolic processes. Studies have shown that chemerin expression in blood and tissues can be clinically evaluated in gastrointestinal diseases. The mechanism of action of chemerin in different disease states deserves further study to explore its possibility as a potential therapeutic target.

## 3. Chemerin in Cancer Cell Processes

### 3.1 Role of chemerin in tumorigenesis and development

Chemerin is expressed differently in different types of tumors such as breast cancer and colorectal cancer and plays an important role in regulating the proliferation, metastasis and invasion of tumor cells. Chemerin receptors are expressed on monocytes/macrophages, T cells, natural killer cells, dendritic cells and neutrophils and are important factors that promote the chemotaxis of various immune cells 26. Chemerin plays important roles in a variety of cancers including melanoma, liver cancer, stomach cancer, and esophageal squamous cell carcinoma, including angiogenesis, adipocyte differentiation, and immune cell migration 27. Previous studies have confirmed that chemerin can increase the invasion of gastric cancer cells, and promote tumor invasion and metastasis by inducing the phosphorylation of p38, extracellular signal-regulated kinases 1 and 2 (ERK1/2) and mitogen-activated protein kinase (MAPK), up-regulating vascular endothelial growth factor (VEGF), matrix metalloproteinase-7 (MMP-7) and interleukin 6 (IL-6) 28, 29. However, chemerin interferes with the interaction of PTEN-CMKLR1 to upregulate the expression of phosphatase and tensin homolog (PTEN) and the activity of phosphatase, resulting in weakened PTEN ubiquitination and decreased phosphorylation of AKT (p-AKT), thus inhibiting the migration, invasion and metastasis of liver cancer cells 30. The combination of chemerin and CMKLR1 can regulate the proliferation, migration and invasion of gastrointestinal and immune cells, as well as cancer-associated cells, and has the potential to become a new target for disease treatment. In addition, chemerin has a strong proinflammatory effect that promotes cancer-related inflammatory responses, further affecting tumor growth. Chemerin promotes the expression of the nucleotide-binding domain, leucine-rich-containing family, and pyrin domain-containing-3 (NLRP3) inflammasome, thereby promoting inflammation and focal shrinkage in diabetic cardiomyopathy rats 31. Chemerin is associated with tumor development, angiogenesis and metastasis 32,33, and serum chemerin content is negatively correlated with tumor size. Blood chemerin levels are higher in patients with different tumors than in healthy controls 34-36. Whether there is a link between circulating chemerin levels and the risk of colorectal cancer needs to be further investigated. Clarifying the role of chemerin in tumors is highly important for further investigations of tumor pathogenesis.

### 3.2 Relationships between chemerin and the tumor immune microenvironment

Chemerin is a protein produced by fat tissue and other cells that plays an important role in immune regulation and inflammation processes. Chemerin may interact with immune cells in the tumor microenvironment and affect immune escape and the antitumor immune response of tumors. Chemerin levels and activity can impact immune cell recruitment and function, in turn influencing tumor cell behavior. mTOR signaling in both immune and tumor cells can modulate their response to chemerin and shape the overall tumor microenvironment 37,38. Chemerin is an important chemotactic agent that induces immune cell recruitment through its receptors CMKLR1, GPR1, and CCRL2, thereby inhibiting tumor growth. Chemerin receptors are expressed on normal and cancer tissues, as well as on various immune cells. Early studies have found that CMKLR1 is expressed only by plasmacytoid dendritic cells (pDCs) in human blood but not by monocytes, neutrophils, eosinophils, lymphocytes, or myeloid DCs 39. The CMKLR1 protein is present not only in all human pDCs but also in approximately 40% of myeloid DCs and the receptor is also expressed in mature DCs. Myeloid-derived suppressor cells are a heterogeneous population of immature myeloid cells that can suppress immune responses, including antitumor immune responses. Chemerin has been shown to be involved in recruiting MDSCs to the tumor microenvironment. These cells can promote tumor progression by suppressing antitumor immune responses, supporting angiogenesis, and facilitating metastasis. In this context, chemerin can contribute to the immunosuppressive tumor microenvironment by recruiting MDSCs 40. Chemerin can also attract tumor-infiltrating lymphocytes (TILs) to tumor tissues, improve the infiltration and activity of immune cells in tumor tissues, and enhance the antitumor immune response. Chemerin also regulates the inflammatory response by recruiting immune cells to the site of inflammation to activate immune cells. Chemerin has been shown to promote inflammation by enhancing the adhesion of macrophages to vascular cell adhesion molecule-1 (VCAM-1) and fibronectin 41. M2 macrophages are a subset of macrophages that are associated with the promotion of tissue repair, angiogenesis, and immuno-suppression. Chemerin has been shown to polarize macrophages toward the M2 phenotype. M2 macrophages within the tumor microenvironment can promote tumor growth, invasion, and metastasis by secreting growth factors, promoting angiogenesis, and suppressing antitumor immune responses. Chemerin-mediated polarization of macrophages toward the M2 phenotype can thus contribute to creating a supportive microenvironment for tumor progression 42. Chemerin can cause immuno-suppression in some cases through the activation of MMP expression, p38 and ERK1/2 MAPK activity and β-arrestin 1, which exert tumor-promoting effects 1, 43-45. In conclusion, the role of chemerin in the tumor immune microenvironment is complex and varied, and may be regulated by multiple factors. However, further understanding of the mechanism of chemerin in tumor immunity is needed.

### 3.3 Potential of Chemerin as a tumor therapeutic target

Studies might explore the use of chemerin as a target for tumor therapy, for example by modulating the chemerin signaling pathway to interfere with tumor growth and metastasis. Chemerin increases the adhesion of mesenchymal stem cells and the adhesion, proliferation and migration of myofibroblasts 46, playing important roles in tissue repair and cancer progression. The Chemerin/ChemR23 axis may modulate the immune response by promoting the pathogenesis of inflammatory diseases and the resolution of acute inflammation through its multiple pathways of action in controlling inflammation, metabolism and carcinogenesis in different organs and systems 47. It has been reported that chemerin can inhibit IL-6 and GM-CSF expression and promote the accumulation of MDSCs, revealing its tumor-inhibitory effect in hepatocellular carcinoma 48. Chemerin is believed to have anti-inflammatory effects that can regulate the inflammatory response and affect the tumor microenvironment 49. By modulating the activation state of immune cells, chemerin may influence tumor development and therapeutic effectiveness. Chemerin may have an inhibitory effect on some tumors. For example, by modulating the ability of tumor cells to proliferate, metastasize, and invade, chemerin could be a potential target in tumor therapy. Chemerin can affect the activation and function of immune cells, possibly affecting tumor development by regulating the recognition and clearance of tumor cells by immune cells 50. Chemerin may have an impact on tumor growth and metastasis by inhibiting angiogenesis; thus, it may be a potential target for tumor therapy. While the potential of chemerin as a tumor therapeutic target is promising, there are challenges that need to be addressed. These include understanding the complex signaling pathways involved, optimizing delivery methods, and ensuring specificity in targeting cancer cells while sparing normal cells. In conclusion, chemerin holds promise as a potential therapeutic target in cancer treatment because of its anti-inflammatory effects, immune-modulatory properties, and ability to inhibit angiogenesis. Further research is needed to fully understand the mechanisms of action of chemerin in cancer and to translate these findings into effective clinical treatments.

### 3.4 Chemerin is associated with tumor-related metabolic diseases

Chemerin expression is closely associated with tumor-related metabolic diseases such as obesity and diabetes. Obesity is a known risk factor for various types of cancer, and chemerin levels are dysregulated in obese individuals. High serum chemerin levels not only induce lipid metabolism and carbohydrate catabolism disorders but also promote an increase in insulin resistance and increase the risk of metabolic disorders 51. Chemerin is a major regulator of fat formation and is closely related to lipid and glucose metabolism; thus, chemerin levels are closely related to obesity and obesity indicators 52,53. In a study of obese children, the associations between chemerin levels and BMI, blood lipid and insulin levels and insulin resistance were determined 54. In patients with type 2 diabetes, higher blood chemerin levels are associated with glycolipid metabolism and inflammatory processes 55, and higher serum chemerin levels are detected in patients with colorectal cancer 56. Therefore, people who are at greater risk for obesity, metabolic disease, or cardiovascular disease have more chemerin. Adipose tissue and the liver are the main organs that produce chemerin 57. Chemerin is strongly associated with glucose and lipid metabolism, obesity, multiple sclerosis, type 2 diabetes, and insulin resistance 58,59. Serum chemerin levels are a useful measure of glucose tolerance. Serum chemerin levels are elevated in patients with tumors, many with hyperglycemia, insulin resistance, and multiple sclerosis. However, the exact relationship between chemerin and tumor-related metabolic diseases is still an area of ongoing research, and the mechanisms underlying this association are not fully understood. Further studies are needed to elucidate the specific role of chemerin in the context of tumor-related metabolic diseases.

## 4. Discussion and Perspectives

Numerous studies have indicated that chemerin is linked to an elevated risk of tumors and tumor-associated metabolic syndrome. This syndrome is further associated with visceral fat obesity, multiple sclerosis, hyperinsulinemia, insulin resistance, low levels of adiponectin, and chronic systemic low-grade inflammation. The underlying causes of these conditions include dysregulation of lipocytokines, insulin resistance, and chronic inflammation. Chemerin levels in the blood of patients with metabolic syndrome or obesity are greater than those in the blood of healthy people, affecting the progression of cancer. To clarify the source of serum chemerin in patients with tumors, more studies need to be conducted. With the continuous deepening and improvement of research, chemerin has broad development prospects in clinical applications. The role of chemerin in the field of tumor research is still being explored and future studies can reveal the specific mechanism of chemerin in tumor biology and provide new ideas and methods for the early diagnosis and treatment of tumors.
